# Entomopathogenicity of Ascomycete Fungus *Cordyceps militaris* on the Cotton Bollworm, *Helicoverpa zea* (Boddie) (Lepidoptera: Noctuidae)

**DOI:** 10.3390/jof9060614

**Published:** 2023-05-26

**Authors:** James P. Glover, Marissa I. Nufer, Omaththage P. Perera, Maribel Portilla, Justin George

**Affiliations:** USDA-ARS Southern Insect Management Research Unit, 141 Experiment Station Rd., P.O. Box 346, Stoneville, MS 38776, USA

**Keywords:** entomopathogenic fungi (EPFs), *Cordyceps militaris*, *Helicoverpa zea*, insect control, lethal concentration 50

## Abstract

This study investigated the exposure of the cotton bollworm, *Helicoverpa zea* (Boddie) (Lepidoptera: Noctuidae) to a novel pathogenic fungal agent historically associated with human medicinal value, a commercial strain of *Cordyceps militaris* ((L.) Fr.) Vuill. (Hypocreales). A series of comparative studies were conducted to evaluate the efficacy of two different exposure methods using four concentrations (*n* × 10^9^, *n* × 10^8^, *n* × 10^7^, *n* × 10^6^) of *C. militaris*, where *n* × 10^9^ provided a concentration of approximately 420 ± 37 spores per mm^2^ with 398 ± 28 viable spores. Survival of cotton bollworms of all stages was not affected by *C. militaris* at any concentration 1 d post-exposure. The greatest reduction in survival and highest sporulation rates were observed primarily on or after 7 d post-exposure for early instars (first and second). Significant declines in the survival of early instars were observed for all concentrations at 7 d, and 95% mortality by 10 d, with the exception of the fifth instars that experienced a less severe reduction in survival (35%) when exposed to any concentrations used in the study. Survival of late instars (third to fifth) ranged from 44% to 68% on day 10, while adult survival was near 99% across the duration of the experiment. The relatively narrow range observed for both the lethal concentration and sporulation of second, third, and fifth instar cotton bollworms exposed to the *C. militaris* strain may demonstrate potential field application for control of larval populations of cotton bollworms.

## 1. Introduction

Significant interest in entomopathogenic organisms, including biopesticides, viruses, bacteria, and fungi capable of parasitization, arresting development, and mortality of insects, have gained tremendous popularity, given the need for environmentally sustainable pest management in modern agriculture [1]. The phenomenon of insect-pathogenic fungal associations and their interactions are well-studied and documented for numerous insect species. Entomopathogenic fungi are routinely used in biocontrol programs to reduce pest populations and insect damage to crops. Biopesticides are biologically based pesticides that utilize toxic molecules harvested from entomopathogenic cultures or live microbes [2]. Traditionally, these practices decrease pest longevity while improving yield and are less harmful to the environment in terms of limited non-target species effects [3]. Pathogenic strains of *Beauveria bassiana* (Bals.-Criv.) Vuill. and *Metarhizium anisopliae* (Metschn.) Sorokīn are among the most widely studied generalist anamorphic fungi encountered in the literature dating from the early 1800s [4]. Anamorphic fungi lack sexual structures and are often able to cause infection in a wide host range and proliferate across many terrestrial ecosystems [5]. The genus *Beauveria* is one of the most common and widely available commercial bioinsecticides or mycopesticides available in a variety of formulations, including wettable powders, liquid concentrates, granules, and seed coatings that help to broaden the targeted host spectrum [6,7,8,9,10,11,12]. In addition, *B. bassiana* offers pest suppression with great success when implemented in integrated pest management strategies, including the Colorado potato beetle, *Leptinotarsa decemlineata* Say (Coleoptera: Chrysomelidae), gypsy moth, *Lymantria dispar* (L.) (Lepidoptera: Erebidae), European corn borer, *Ostrinia nubilalis* (Hübner) (Lepidoptera: Crambidae), and whiteflies, *Bemisia tabaci* (Gennadius) (Hemiptera: Aleyrodidae) [12,13,14,15,16].

Studies with specialized teleomorphic species of entomopathogenic fungi are less frequently encountered in the literature [5]. *Cordyceps militaris* (CM) (Hypocreales: Cordycipitaceae) is a species of the genus *Cordyceps*, comprised of more than 600 insect-pathogenic species and has been a source of biologically active substances extensively studied in biotechnology, nutraceuticals, and pharmacological applications [17,18]. *Cordyceps* spp. have distinct and unique growth characteristics and exist in the natural environment as entomopathogenic fungi that commonly attack and colonize the larvae and pupae of *Thitarodes* spp. and other lepidopteran spp. *Cordyceps militaris* is unique, requiring two mating compatible strains to produce perithecial stromata or fruiting bodies [19,20]. Caterpillars infected with *Cordyceps* spp., commonly known as the orange caterpillar fungus, Scarlet Caterpillar club, or winter worm-summer grass, contain carotenoids which are responsible for the yellow-orange color commonly observed on fruiting bodies erupting from insect cadavers. Carotenoids are light-harvesting pigments produced by plants, algae, and fungi that act as antioxidants in humans. The genus *Cordyceps* has an extensive history of use in health and medicinal uses in traditional Chinese medicine and common dietary supplements [17,21,22,23].

The bollworm *Helicoverpa zea* (Boddie) (Lepidoptera: Noctuidae) is found in almost all regions of North America and feeds on more than 500 plant species [24]. The bollworm is considered one of the most devastating insect pests in cotton across the United States [25]. Significant yield losses and control costs in excess of $25 million annually are attributed to bollworms [26,27]. The bollworm is highly mobile and feeds on many economically important food and fiber crops, including maize (*Zea mays* L.), cotton (*Gossypium hirsutum* L.), and soybean (*Glycine max* L.) [28]. In the Southeastern U.S., bollworm populations are multivoltine and known to complete four or more generations per year [24]. Developing larvae cause significant economic damage to several commercial row crops, including whorl feeding and ear tip damage in corn and significant feeding and tunneling damage to developing cotton fruiting structures.

Here, we conducted a series of comparative studies to evaluate the efficacy of two different exposure methods (topical and contact) to four concentrations of spores from a specialized teleomorphic entomopathogenic fungus species, *C. militaris,* on the survival, pupation, mortality, concentration–mortality values (LC_50_), and concentration-Sporulation response (LS_50_) estimates for all life stages of the cotton bollworm.

## 2. Materials and Methods

### 2.1. Insect Source and Rearing

The cotton bollworm colony used in experiments was originally field collected in late spring 2022 from several counties in the Mississippi Delta from Crimson clover, *Trifolium incarnatum* L., using a 38.1 cm (15″) diameter sweep net. The research colony was maintained on an artificial wheat-germ-based solid diet developed for Lepidoptera for four successive generations at USDA-ARS, Insect Management Research Unit, Stoneville, MS, USA. Insect colonies were reared at 28 °C in environmental chambers with a photoperiod of 16:8 h (L:D) and 65% RH. Neonates and all larval instars were 1–2 d old, with an assumed equal proportion of males to females, were used for experiments.

### 2.2. Identity of Fungal Isolate

#### Mating Type Determination

*Cordyceps militaris* isolate Tf-4, originating from the Appalachian Mountains, USA, were used in bioassays (Terrestrial fungi, Smurfville, MI, USA). DNA from 3 d old single spore cultures cultivated on potato dextrose agar (PDA) media and held in an environmental chamber (21 °C, 65% RH, dark 0/24 h L/D photoperiod) was extracted. A fresh fungal agar plug was removed from a plate and inserted directly into a 1.5 mL tube containing a solution of 188 μL Tissue and Cell Lysis Solution (Lucigen Corp., Middleton, WI, USA), and 4 μL of Proteinase K (Invitrogen, Waltham, MA, USA), then ground down using a test-tube specific pestle. Samples were then incubated at 65 °C for one hour, 10 μL RNase was added, mixed into each sample and incubated at 37 °C for 30 min. Samples were then placed on ice for five minutes before 120 μL of MPC Protein Precipitation Reagent (Lucigen Corp.) was added to each sample, shaken vigorously, then centrifuged for 15 min at 16,100× *g* at 4 °C. The supernatant from each sample was then carefully transferred to a new tube containing an equal amount of 100% isopropanol and inverted several times to mix. Tubes were placed in the 4 °C centrifuge at 16,100× *g* for 20 min leaving behind a DNA pellet that was washed twice with 400 μL of 70% ethanol. After the second ethanol wash, samples were spun down briefly, and any traces of ethanol were aspirated to ensure the purity of the remaining DNA pellet. The pellet was dried for five minutes and dissolved in 35 μL of 10 mM Tris-HCl (=pH 8.0). Samples were quantified using a Nanodrop C2000 instrument (Thermo Fisher Scientific, Waltham, MA, USA).

Mating type loci *MAT-α (MAT1-1)* and *MAT-HMG (MAT1-2)* were used to identify idiomorphs of *C. militaris* by amplifying DNA isolated from single-spore colonies with three pairs of locus-specific primers. PCR primer pairs MAT1-1-1-F/R (5′-ATGGAACACAGATCGAGCGACAC-3′/5′-ATATACCTTCGCGATCATTGCCCAG-3′) and MAT1-1-2 F/R (5′-TTCAGCTTCAGTCCGTTCTGGACA-3′/5′-GGCAGACATCGTACCTGGTCAAAT-3′) were used to identify the *MAT-α* mating type. The *MAT-HMG* mating type was identified using the primer pair MAT1-2-1-F/R (5′-ATGGATCTGCAACTGGATCGGACCA-3′/5′ CTACATGATTGACTCCGGGCTCATTG-3′) [29]. PCR amplification of 35 μL volume was set up using the following reagents: 1× concentrations Taq (Mg-Free) reaction buffer (New England Biolabs, Ipswich, MA, USA), 1.5 mM MgCl2 (NEB), 200 M dNTP’s, 0.2 μM forward primer, 0.2 μM reverse primer, 1.0 μL of template DNA (concentration varies based on sample), 0.875 units of *Taq* (New England Biolabs, Ipswich, MA, USA), and nuclease-free water to achieve 35 μL total volume (Invitrogen, Waltham, MA, USA). The reaction was performed using a PTC-200 Peltier Thermal Cycler (MJ Research, St. Bruno, QC, Canada) under the following conditions: one cycle of 95 °C for 30 s followed by 95 °C for 10 s, at 56 °C for 15 s, and 72 °C for 90 s for 35 cycles, with a holding temperature of 0 °C at the end. Samples were then run on a 1% agarose gel at 100 volts for 50 min. The mating type of each single-spore culture was determined based on the amplicon size, as shown in previous publications [19,30].

### 2.3. Spore Cultivation

*Cordyceps militaris* was produced and stored at −80 °C at the Southern Insect Management Research Unit (SIMRU), Stoneville, MS, USA. Spore powder production was accomplished by utilizing a biphasic culture system. A single germinated spore was collected from each mating type with a sterile inoculation loop and suspended in 1000 mL of potato dextrose broth amended with (2.5 g/L Yeast Extract and 2.5 g/L casein and agitated in a shaker (Incubator Shaker Series Excella E25, New Brunswick Scientific Co., Inc., Edison, NJ, USA) for five days at 27 °C to produce the inoculum used in bioassays. Twelve plastic bags (560 mm × 385 mm × 225 mm) that contained 1000 g of brown rice and 800 mL of water were autoclaved and prepared for the 5 d inoculum. Aliquots of 50 mL of the inoculum (suspended *C. militaris*-PD broth) were injected into each bag and allowed to fully colonize the rice in an environmental chamber (27 °C, 50% RH, dark 0/24 h L/D photoperiod) for seven to ten days. Inoculum bags were flipped every 24 h to facilitate complete colonization allowing hyphal growth to disperse throughout the rice substrate fully. The conidial substrate was allowed to dry in paper bags (30 cm × 17 cm × 43 cm Barrel, Kraft) for seven to ten days until sufficiently dry (a_w_ ≤ 0.3). Graded sieves were hand shaken (Grainger, Sieve SS Frame 8, SS Mesh # 15, 30, and 100) to separate the conidia from the dried rice. Harvested spores were plated on PDA, allowed to grow for 24 h and examined for spore germination, and enumerated (spores mm^2^). A final concentration of 5.02 × 10^9^ spores per mL was obtained using a hemocytometer 0.1 mm deep (Hausser-Bright Line, Horsham, PA, USA) from diluting 0.5 g of harvested spores containing 3.1 × 10^11^ spores suspended in 50 mL of 0.04% Tween-80 (Sigma-Aldrich P8074). Disposable glass microscope cover slip slides (Fisherbrand™ Disposable Cover Slips, Thermo Fisher Scientific, Waltham, MA, USA) were sprayed with suspensions of conidia using a handheld sprayer that covered an area of 38.5 mm^2^ in diameter. The concentration (spores per mm^2^) was determined by counting deposited spores, and the procedure was repeated five times using the final concentration (*n* × 10^9^) (*n* = diluted harvested spore powder of 5.02). The concentration of spores (*n* × 10^8^, *n* × 10^7^, *n* × 10^6^) was estimated and based on dilution of *n* × 10^9^ concentration. The number of spores applied was adjusted for viability across all concentrations tested. Aliquots of 6 mL suspensions (*n* × 10^9^) provided a concentration of approximately 420 ± 37 viable spores per mm^2^ with 398 ± 28 viable spores (95% viability).

### 2.4. Exposure Protocol

Cotton bollworms were treated with serial dilutions of *C. militaris* (*n* × 10^9^, *n* × 10^8^, *n* × 10^7^, *n* × 10^6^) spores (see above). Mortality, pupation, and sporulation were recorded daily for 10 d. Bioassays were conducted in environmental chambers constantly held at 28 °C with a photoperiod of 16:8 h (L:D) and 65% RH. Each treatment combination (concentration × exposure method) was replicated four times for all developmental stages (324 individuals for each instar, pupae, and adults, respectively). Concentrations of *C. militaris* and the water control (*n* × 10^9^, *n* × 10^8^, *n* × 10^7^, *n* × 10^6^ spores/mL) were applied in a 6 mL spray volume. Individuals were observed daily for 10 consecutive days under a light microscope by removing the plastic lid. Larvae were scored deceased if there was no observable movement when prodded with a natural hair paint brush or alive if the movement was detected. Larvae were maintained within the original diet cup, and mycosis was determined by daily observations of individuals for the first detection of external hyphal growth. Instars were determined based on the number of shed head capsules observed in the diet cup.

#### 2.4.1. Direct Exposure: Spores Applied Directly to Insect Cuticle

Aliquots of 6 mL of each treatment suspension and water control were sprayed topically to the insect cuticle for each repetition (30 individuals/repetition/treatment) (1st, 2nd, 3rd, 4th, 5th instars, pupae, and adults). A non-autoclaved, wheat-germ-based artificial solid diet was used for this experiment (see above). Test concentrations were applied with individual spray bottles for each concentration to prevent cross-contamination. Groups of treated insects were placed in observation cages (Popup rearing cages 1466 PB) (130 cm × 130 cm × 130 cm, Bugdorm^TM^, BioQuip Products, Rancho Dominguez, CA, USA) and allowed to air dry. A freshly sanitized cage was used for each replicate and treatment combination to prevent cross-contamination. Dried insects were paint brushed individually into plastic cups containing a standard heliothine lab-made diet. Adults were placed in cardboard cups (32 cm × 32 cm × 87 cm) and were supplemented with 5% sucrose solution delivered on cotton balls. Insects were sprayed on an absorbent filter paper and discarded in between each treatment, which allowed insects to move freely and prevented accumulation of conidial solution and potential drowning of neonates and young instars.

#### 2.4.2. Contact Exposure: Treated Corn Leaf Tissue

Similarly, for direct exposure, 6 mL of each spore concentration were sprayed onto freshly excised non-Bt corn leaf tissues of similar age, and a water-sprayed control from greenhouse plants grown free of insect injury and synthetic insecticides was used. (30 individuals/repetition/treatment) (1st, 2nd, 3rd, 4th, 5th instars). Corn tissue sections (25 cm × 25 cm × 25 cm) were placed in a disposable ice cream cardboard bucket (56 cm in diameter) with the base covered with filter paper and treated topically. Sprayed corn tissues were placed under a fume hood and allowed to dry before placing them in diet cups and introducing them to the insects.

### 2.5. Statistical Analysis

Experiments were analyzed using PROC GLIMMIX [29] (4 dilutions × 2 exposure methods; contact with treated tissues and topically applied to insects plus a water control). Each treatment combination was repeated six times. A one-way ANOVA was used to analyze mortality and sporulation using SAS 9.4 (SAS Institute, Cary, NC, USA). A Tukey’s post hoc HSD test was used to compare means among treatments [29]. The PROC PROBIT procedure in SAS using log base 10 of the concentrations was used to analyze lethal concentration and lethal sporulation. Nonparametric estimates of survival were used to compare treatments using PROC LIFETEST-SAS, and mortality was corrected using Abbot’s formula. Confidence intervals were calculated using the method of Robertson and Priestler [31].

## 3. Results

### 3.1. Verification of Fungal Isolate Identity

**Figure 1:**
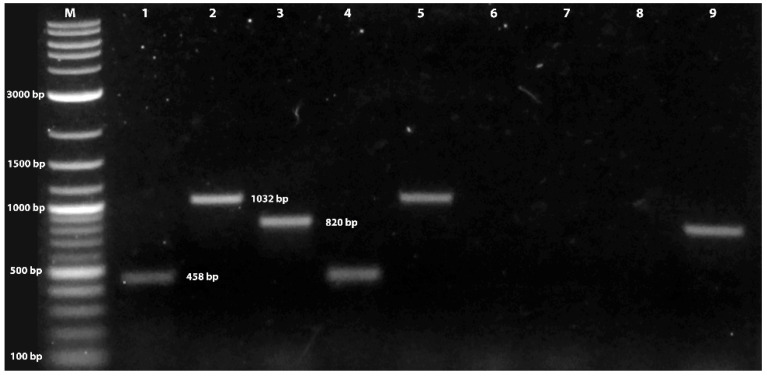
An agarose gel of amplicons produced from *Cordyceps militaris* hybrid (lanes 1–3), MAT-α (lanes 4–6), and MAT-HMG (lanes 7–9) mating type DNA by primer pairs MAT1-1-1F/R (lanes 1, 4, and 7), MAT1-1-2F/R (lanes 2, 5, and 8), and MAT1-2-1F/R (lanes 3, 6, and 9), respectively. Amplicons produced from all three primer pairs are present in the hybrid isolate (lanes 1–3). Only the amplicons specific to each mating type idiomorph were produced with DNA from MAT-α (lanes 4, 5) and MAT-HMG (lane 9). M = 1 k Plus DNA ladder (New England Biolabs, Ipswich, MA, USA).

Cloned amplicons yielded nucleotide sequences; MAT1-1-1, length: 458 bp (nucleotide identities: 451/455) (KP721272), MAT1-1-2, length: 1032 bp (nucleotide identities 967/976) (AB194982), and MAT1-2-1, length: 820 bp, (nucleotide identities: 346/355) (AB084257). Searches of the BLAST databases using these nucleotides revealed the highest nucleotide identity (between 97 and 99%) to mating type genes MAT1-1 and MAT1-2 of *C. militaris* isolate (Figure 1). The nucleotide sequence of the amplicons of two mating types was submitted to GeneBank under accession numbers OR039169–OR039173.

### 3.2. Larval, Pupal, and Adult Mortality

**Figure 2:**
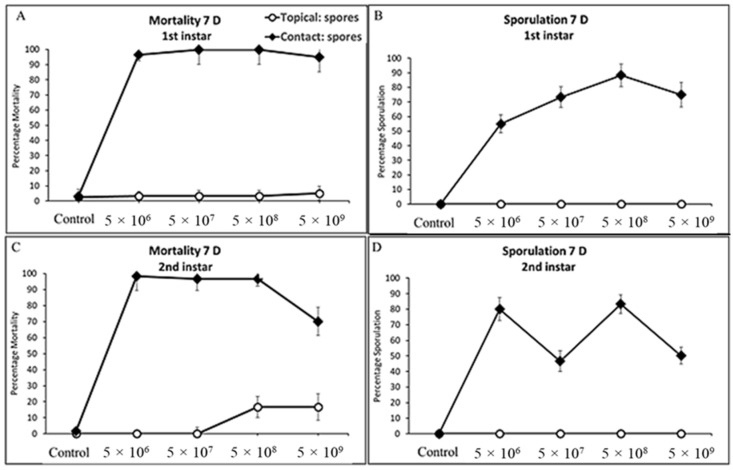
Cumulative mortality and sporulation percentage for first (**A**,**B**) and second (**C**,**D**) instars of cotton bollworm exposed to (CM) by contact (treated leaf tissue) and direct spray (topical) at four concentrations.

**Figure 3:**
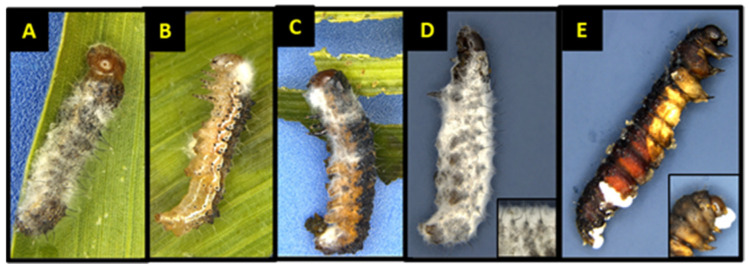
Cotton bollworm larval instars infected with (CM) spores after exposure to sprayed corn leaf tissue. (**A**) first instar; (**B**) second instar; (**C**) third instar; (**D**) fourth instar, inset image: mycosed larvae 6 days after exposure; (**E**) fifth instar, inset image: mycosed larvae with hyphae erupting from insect mouth and anus 10 d after exposure.

**Figure 4:**
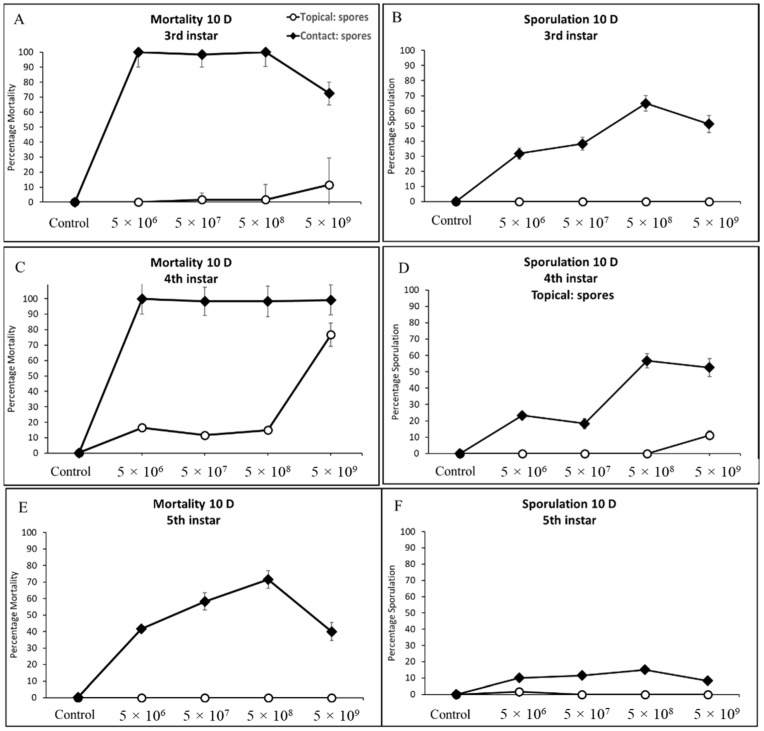
Cumulative mortality and sporulation percentage for third (**A**,**B**), fourth (**C**,**D**), and fifth (**E**,**F**) instars of cotton bollworm exposed to (CM) by contact (treated leaf tissue), and direct spray (topical) at four concentrations.

Significant differences in mortality were detected for all developmental stages of the cotton bollworm when exposed to spores through contact and consumption of treated corn leaf tissues. Survival analysis indicated significant differences in mortality across concentrations for life stage and exposure method by day 7 for first and second instars and day 10 for all other larval developmental stages, with the exception of fifth instars and adults treated topically irrespective of concentration (Log-Rank X^2^ = 8.44, df = 3, *p* = 0.305; X^2^ = 11.31, df = 1, *p* = 0.428) (Table 1) (Figure 2, Figure 3 and Figure 4). A decline in survival was not affected by any spore concentration used in this series of experiments 24 h post for any life stage assayed, apart from first instars, with 18% mortality plateauing at day 10 (>95%). The highest mortality observed in this study was on day 7 for first and second instars and day 10 for third, fourth, and fifth instar cotton bollworms. Infected cadavers began to sporulate irrespective of concentration, ranging from 5 d to 7 d and plateauing on day 10 after exposure to treated corn leaf tissues. Adults topically treated regardless of concentration experienced minimal mortality (<17%), and no sporulation was observed on treated adults 10 d and 20 d post-application. Regardless of concentration or exposure method (topical or contact), mortality increased from day 5 through day 10 in this study (Figure 2, Figure 3 and Figure 4).

### 3.3. Larval Mortality and Sporulation

#### 3.3.1. First and Second Instars Cotton Bollworm

**Figure 5:**
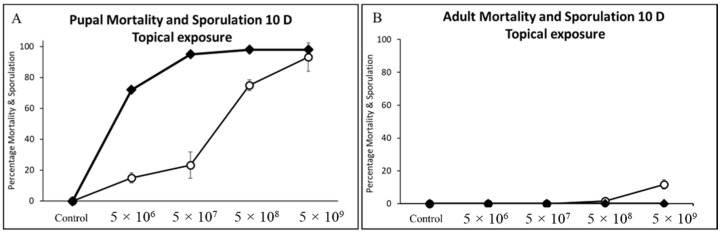
Cumulative mortality and sporulation percentage for (**A**) pupae and (**B**) adult cotton bollworm exposed to CM spores by topical application (applied directly to insect cuticle) at four concentrations.

Spore concentrations were highly pathogenic and caused significant mortality when applied to corn leaf tissues and fed to cotton bollworms, leading to varying levels of mycosis and sporulated individuals (*F* = 556.32, df = 3, 324, *p* < 0.0001; *F* = 116.67, df = 3, 324, *p* < 0.0001) and (*F* = 51.44, df = 3, 324, *p* < 0.0001; *F* = 35.73, df = 3, 24, *p* < 0.0001) for first and second instars, respectively (Figure 5A,B). Exposure of first and second instar larvae of cotton bollworm to spores topically, irrespective of concentration, had significantly less mortality (*p* > 0.05) with <12% and <16% (Figure 2A,C). Although not statistically different, higher rates of mortality and sporulation were observed for first and second instars when exposed to spores through contact with treated tissues at the *n* × 10^8^ concentration. Significant differences or interactions of mortality and sporulation based on spore concentration were not detected for either larval developmental stage at day three or five (*p* > 0.05). Control treatments consisting of water by contact (water sprayed tissue) and direct spray never resulted in sporulation of individuals. Exuviae were frequently observed with initial colonization with hyphae on all larval stages at any concentration by both contact and the direct spray of both spores and hyphal suspensions.

#### 3.3.2. Third, Fourth, and Fifth Instar Cotton Bollworm

Similarly, contact treatments (sprayed corn leaf tissue) of all concentrations when offered to cotton bollworms caused infection leading to significant mortality and sporulation (*F* = 66.48, df = 3, 324, *p* < 0.0001; *F* = 10.18, df = 3, 24, *p* < 0.0002), (*F* = 18.08, df = 3, 24, *p* < 0.0001; *F* = 7.33, df = 3, 24, *p* < 0.0012), and (*F* = 51.99, df = 3, 24, *p* < 0.0001; *F* = 9.68, df = 3, 24, *p* < 0.0002) for third, fourth, and fifth instars, respectively (Figure 3C–E). Sporulation of water control treatments either by contact or direct spray was not detected anytime across this study. Exposure of third and fourth instars of cotton bollworm to spores topically, irrespective of concentration, had markedly high mortality with >95% (Figure 4A,C). Fourth instars showed significant mortality for the highest spore concentration when applied topically >75%; however, sporulation remained low (<13%). Fifth instars experienced significantly more variation in mortality when compared to other instars at the same concentrations ranging from 42–71% (Figure 4E). Sporulation across third and fourth instars was highly variable, with rates ranging from 33% up to 62%. In comparison, fifth instars experienced <16% sporulation when exposed to contact with treated leaf tissues. Even though statistically non-significant, ecologically relevant differences in mortality and sporulation were observed for third and fourth instar sporulation through contact with treated tissues, and fifth instar mortality >70% at the *n* × 10^8^ concentration compared to others tested.

### 3.4. Pupal and Adult Mortality and Sporulation

**Figure 6:**
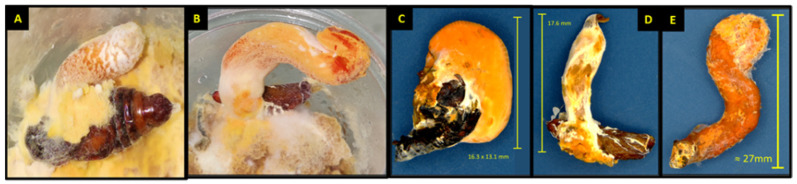
Range of stroma growth on cotton bollworm pupae infected with (CM) spores after topical exposure. (**A**) pupae with perithecial stroma (55 d); (**B**) pupae with developing stroma stalk (45 d); (**C**) pupae with large stroma erupting from the thoracic region; (**D**) pupae with stroma erupting from the abdomen; (**E**) characteristic stroma excised from pupae exhibiting orange-yellow pigmentation (60 d).

**Figure 7:**
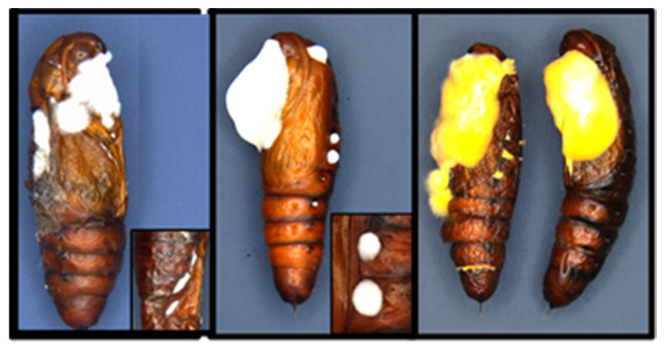
Cotton bollworm pupae (2 d) infected with CM spores after topical exposure. (**Left** and **middle** panel) infected pupae 10 d after exposure with external hyphal growth, inset image: Fungal eruptions from spiracles; (**Right** panel) infected pupae 20 d post-exposure with characteristic orange-yellow external masses.

**Figure 8:**
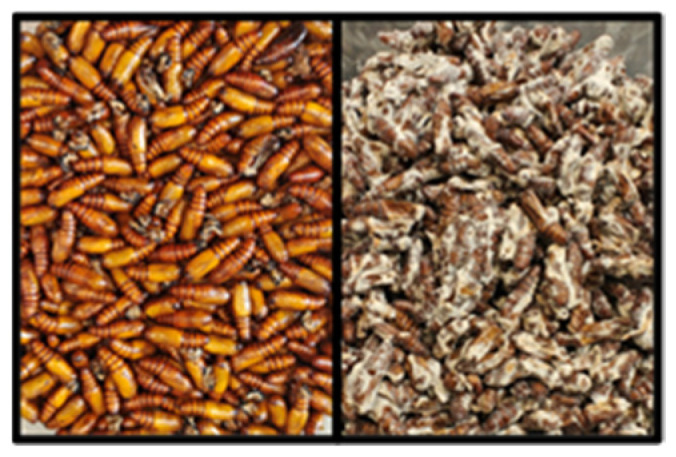
Cotton bollworm pupae (2 d). (**Left** panel) uninfected healthy control pupae 7 d after topical exposure to water; (**Right** panel) infected pupae 15 d after topical exposure to CM spores exhibiting external hyphal growth.

Teleomorphic fruiting bodies of *C. militaris* erupted from cotton bollworm pupae on <10% of pupae topically treated with any spore concentration (Figure 6). All concentrations tested when applied topically to the pupae were pathogenic and caused significant mortality and sporulation (*F* = 24.55, df = 1, 24, *p* ≤ 0.0001; *F* = 17.55, df = 1, 24, *p* ≤ 0.0003) (Figure 6, Figure 7 and Figure 8). Statistically significant differences were detected for cotton bollworm pupal mortality (>76%) when exposed to *n* × 10^8^ concentration compared to others tested (*F* = 5.73, df = 1, 24, *p* ≤ 0.0249). However, no significant decline in mortality or sporulation was detected for adults topically applied with spores. Adult sporulation was never observed from the topical treatment of spores at any concentration. Significant differences or interactions of mortality and sporulation based on spore concentration were not detected for adults topically treated (*p* > 0.05).

### 3.5. Lethal Morality and Response of Cotton Bollworm Instars

The LC_50_ values (spores/mm^2^) determined by probit analysis indicated considerable variability across the life stages of cotton bollworms and the exposure method (Table 2 and Table 3). The probit model for cotton bollworm larval instars exposed by contact (treated corn leaf tissue) with spores of *C. militaris* produced a significant fit of the data for concentrations among instars, with the exception of first and fourth instars of cotton bollworm that experienced >98% mortality at any concentration (Table 2). A comparison of dose–mortality values indicated significant differences between the life stages of the cotton bollworm to the *C. militaris* strain. The LC_50_ values showed that second, third, and fifth instar bollworms exhibit similar susceptibility to *C. militaris,* given the narrow range (Table 2). The probit model for instars exposed to (CM) spores by topical application (directly to insect cuticle) produced a significant fit of the data for concentrations among pupae and instars, with the exception of first, second, fifth instars, and adults of cotton bollworm that experienced <17% mortality at the highest concentrations (Table 3). The LC_50_ values showed that third and fourth instar cotton bollworms are similarly susceptible to *C. militaris,* given the narrow range. Pupae were highly susceptible to infection and mortality with exposure to significantly fewer spores (LC_50_) of 2.01 spores than instars (Table 3). All remaining topical treatment combinations for instars and adults resulted in no significant relationship, given the low rates of mortality observed.

### 3.6. Cordyceps militaris Concentration-Sporulation Response (LS_50_) on Cotton Bollworm Instars

The regression analysis performed with sporulation percentage for each concentration tested showed a linear relationship as spore concentration increased. A similar trend was observed for sporulation of cadavers when exposed by contact to treated tissues; r^2^ values ranged from 0.96, 0.87, 0.79, 0.32 for *n* × 10^9^, *n* × 10^8^, *n* × 10^7^, *n* × 10^6^ spores/mL, respectively. Individuals that were exposed to treated tissues at any concentrations sporulated at significantly higher rates when the exposure method was through contact with treated tissues compared with insects treated with spores topically (*F* = 116.67, df = 3, 324, *p* < 0.0001), (*F* = 35.73, df = 3, 324, *p* < 0.0001), (*F* = 10.18, df = 3, 324, *p* < 0.0002), (*F* = 7.33, df = 3, 324, *p* < 0.0012), (*F* = 9.68, df = 3, 324, *p* < 0.0002), for first, second, third, fourth, and pupae, respectively. Adults, regardless of concentration, never sporulated during this experiment (*p* > 0.05). Analyses of concentration-sporulation response by developmental stage and exposure method to *C. militaris* isolate showed moderate variability in concentration–sporulation response among young instars which encountered spores through contact with treated tissues, compared to topical exposure having negligible effects on sporulation response (Table 4). Concentration ratios of spores for sporulation response (LS_50_) were all within a similar range, with the exception of fifth instars, for which sporulation never exceeded >17% for any concentration tested, and fourth instars, which sporulated with exposure to significantly less concentration and (LS_50_) of 2.14 spores (Table 4). The *C. militaris* isolate used in this series of experiments was significantly effective in causing sporulation and mycosis at any concentration tested for larval instars, but not the adults.

## 4. Discussion

This is the first study to assess the potential insecticidal effects of the specialized ascomycete fungus *C. militaris* on the cotton bollworm. Significant differences observed in the mortality-contrasting exposure method indicated that larval developmental stages of cotton bollworms could readily acquire lethal concentrations of conidia from the consumption of treated tissues. Data from these experiments demonstrated that infectivity and mortality (>97%) associated with *C. militaris* fungal isolate occurred primarily through contact and consumption of treated corn leaf tissues. Fourth instar cotton bollworms were the only larval stage that experienced a significant decline in mortality when sprayed directly (topically) at the highest concentration having <14% sporulation. Pupae were highly vulnerable to infection. The highest concentration of *C. militaris* used in this study (*n* × 10^9^) was consistently enough to obtain >97% mortality and (*n* × 10^8^) achieving nearly 60% sporulation in cotton bollworms under laboratory conditions 10 days after exposure through contact and consumption of treated tissues in this study.

The relatively narrow range of LC_50_ 2.01–11.82 and LS_50_ of 2.14–10.36 spores/mm^2^, respectively, observed for *C. militaris* demonstrates that a relatively low concentration and few spores caused infection leading to high rates of mortality. In this study, we utilized both mating types for bioassays; however, this species has been reported to produce stroma or fruiting bodies without a mature perithecial strain with a single mating type and may possess insecticidal activity individually and warrant further study [32]. Results from bioassays suggest the potential field application of *C. militaris* spores as a pest management tool for cotton bollworms. While *C. militaris* may not currently be commercially viable given the complexity of the biphasic growth system, it may have applications alongside traditional insect management strategies, such as use in non-crop alternative hosts to reduce populations of cotton bollworm. Traditional biological management strategies may be enhanced when combined or used in rotation with *C. militaris.* This is the first study to directly measure the pathogenicity in terms of mortality and sporulation of *C. militaris* in all developmental stages of cotton bollworm. The authors found no similar experiments to draw a comparison, with the exception of the silkworm *Bombyx mori* (Linnaeus) (Lepidoptera: Bombycidae), used as the substrate to produce medicinal strains of *C. militaris* [33,34]. All experiments were conducted under laboratory conditions that are highly controlled and favorable to the growth of *C. militaris*. Future studies are needed to examine the pathogenicity of this strain on various insect pests commonly associated with foliar feeding in corn and cotton.

## Figures and Tables

**Table 1:** Test of Equality LIFETEST for developmental stages of cotton bollworm exposed to four different concentrations of *Cordyceps militaris*. Survival was scored for 10 d post-exposure.

Life Stages	Contact-Treated Leaf Tissue			Topical-Spray Application		
	X^2^	*DF*	*p* > X^2^	X^2^	*DF*	*p* > X^2^
1st instar	13.411	1	0.003	0.445	1	0.104
2nd instar	10.045	1	0.002	19.000	1	0.001
3rd instar	19.602	1	0.001	1.583	1	0.048
4th instar	14.032	1	0.005	2.671	1	0.002
5th instar	6.105	1	0.028	8.444	1	0.305
Pupae	-	-	-	16.333	1	0.001
Adult	-	-	-	11.314	1	0.428

**Table 2:** Lethal mortality-response (LC_50_) of cotton bollworm larval instars exposed by contact (treated corn leaf tissue) with spores of *Cordyceps militaris*.

Larval Instars	Concentration Response (Spores/mm^2^)						
	*n*	Slope ± SE	LC_50_ (95% CI)	Probit Trend			
				Test for Slope		Test for GoF ^1^	
				X^2^	*p* > X^2^	X^2^	*p* > X^2^
1st instar	324	-	-	0.77	0.389	2.565	0.0769
2nd instar	324	5.08 ± 1.01	10.13 (9.54–11.47)	29.52	0.0001	3.524	0.0295
3rd instar	324	8.53 ± 2.15	9.80 (9.13–11.53)	15.06	0.0001	5.026	0.0066
4th instar	324	-	-	0.13	0.714	0.636	0.5292
5th instar	324	3.91 ± 1.27	13.61 (11.19–16.53)	8.21	0.0148	8.011	0.0003

Mortality was scored at 10 d post-exposure. ^1^ Test for Goodness of Fit (GoF) indicates an error from Probit trend *p* > 0.05.

**Table 3:** Lethal mortality-response (LC_50_) of cotton bollworm topically treated with spores of *Cordyceps militaris*.

Stage	Concentration Response (Spores/mm^2^)						
	*n*	Slope ± SE	LC_50_ (95% CI)	Probit Trend			
				Test for Slope		Test for GoF	
				X^2^	*p* > X^2^	X^2^	*p* > X^2^
1st instar	324	-	-	0.16	0.6884	0.12	0.9406
2nd instar	324	-	-	0.54	0.4602	5.53	0.0039
3rd instar	324	3.27 ± 1.02	8.73 (8.35–9.89)	14.23	0.0002	4.23	0.0146
4th instar	324	6.16 ± 2.13	11.82 (10.05–22.07)	11.02	0.0091	1.45	0.2333
5th instar	324	-	-	-	-	-	-
Pupae	324	7.45 ± 1.47	4.01 (3.94–5.04)	6.77	0.0093	7.52	0.0233
Adult	324	-	-	2.15	0.1426	9.49	0.0001

Mortality was scored at 10 d post-exposure.

**Table 4:** Lethal Sporulation-response (LS_50_) of cotton bollworms treated by contact (treated diet) and direct topical spray with spores of *Cordyceps militaris*.

Stage				Concentration Response (Spores/mm^2^)				
	Exposure	*n*	Slope ± SE	LS_50_ (95% CI)	Probit Trend			
					Test for Slope ^1^		Test for GoF	
					X^2^	*p* > X^2^	X^2^	*p* > X^2^
1st Instar	Contact ^2^	324	3.14 ± 0.95	6.52 (4.54–11.73)	13.16	0.0084	3.19	0.0225
	Topical ^3^	324	-	-	1.76	0.1944	4.24	0.0145
2nd Instar	Contact	324	1.33 ± 0.65	7.84 (5.42–16.75)	25.22	0.0001	13.29	0.0001
	Topical	324	-	-	1.72	0.1897	9.49	0.0001
3rd Instar	Contact	324	1.41 ± 0.53	9.85 (3.14–11.25)	6.43	0.0112	14.21	0.0002
	Topical	324	-	-	1.61	0.2041	4.01	0.0183
4th Instar	Contact	324	2.36 ± 0.64	2.14 (1.74–2.26)	12.23	0.0023	9.54	0.0005
	Topical	324	4.45 ± 1.11	10.33 (9.42–13.17)	17.01	0.0001	5.17	0.0057
5th Instar	Contact	324	-	-	0.56	0.4536	4.80	0.0082
	Topical	324	-	-	0.51	0.9741	0.73	0.4823
Pupae	Topical	324	4.07 ± 0.49	9.52 (8.18–11.19)	11.44	0.0003	9.18	0.0035

(-) Indicates *p* > 0.05 regression. ^1^ Test for slope significance indicates dose affects mortality. ^2^ Topical exposure. ^3^ Exposure to spores via treated corn tissues.

## Data Availability

Not applicable.
